# Correlation between Intraprostatic PSMA Uptake and MRI PI-RADS of [^68^Ga]Ga-PSMA-11 PET/MRI in Patients with Prostate Cancer: Comparison of PI-RADS Version 2.0 and PI-RADS Version 2.1

**DOI:** 10.3390/cancers12123523

**Published:** 2020-11-26

**Authors:** Jing Zhao, Dilyana B. Mangarova, Julia Brangsch, Avan Kader, Bernd Hamm, Winfried Brenner, Marcus R. Makowski

**Affiliations:** 1 Institute of Radiology and Nuclear Medicine, Charité–Universitätsmedizin Berlin, Corporate Member of Freie Universität Berlin, Humboldt-Universität zu Berlin, and Berlin Institute of Health, Charitéplatz 1, 10117 Berlin, Germany; dilyana.mangarova@charite.de (D.B.M.); julia.brangsch@charite.de (J.B.); avan.kader@charite.de (A.K.); bernd.hamm@charite.de (B.H.); marcus.makowski@charite.de (M.R.M.); 2 Department of Veterinary Medicine, Institute of Veterinary Pathology, Freie Universität Berlin, Robert-von-Ostertag-Str. 15, Building 12, 14163 Berlin, Germany; 3 Department of Biology, Chemistry and Pharmacy, Institute of Biology, Freie Universität Berlin, Königin-Luise-Str. 1-3, 14195 Berlin, Germany; 4 Department of Nuclear Medicine, Charité–Universitätsmedizin Berlin, Corporate Member of Freie Universität Berlin, Humboldt-Universität zu Berlin, and Berlin Institute of Health, Augustenburger Platz 1, 13353 Berlin, Germany; winfried.brenner@charite.de; 5 Department of Diagnostic and Interventional Radiology, Klinikum rechts der Isar, Technische Universität München, Ismaninger Str. 22, 81675 Munich, Germany

**Keywords:** prostate cancer, multiparametric MRI, PSMA, [^68^Ga]Ga-PSMA-11 PET/MRI, SUVmax, PSA, molecular imaging, PI-RADS 2.1

## Abstract

**Simple Summary:**

The newest Prostate Imaging Reporting and Data System (PI-RADS) version 2.1, was published in 2019. There are a few variations of the new standard, which will change prostate lesions’ classification rules. Our study aims to analyze the pattern change of lesion positron emission tomography (PET) standardized uptake values maximum (SUVmax) distribution under PI-RADS V2.1, compared with PI-RADS V2.0. Moreover, we studied the correlation between prostate-specific membrane antigen (PSMA) SUVmax and magnetic resonance imaging (MRI) PI-RADS. So far, there is no article reporting the effect of the newest PI-RADS on [^68^Ga]Ga-PSMA-11 PET/MRI. We did a thorough analysis, including two subgroups, peripheral zone, transitional zone, and 215 lesions. We analyzed the impact of each variation of PI-RADS one by one.

**Abstract:**

Purpose: We aimed to evaluate the correlation between PSMA uptake and magnetic resonance imaging (MRI) PI-RADS of simultaneous [^68^Ga]Ga-PSMA-11 PET/MRI regarding PI-RADS version 2.0 and 2.1 respectively and compared the difference between these two versions. Materials and Methods: We retrospectively analyzed a total of forty-six patients with biopsy-proven prostate cancer who underwent simultaneous [^68^Ga]Ga-PSMA-11 PET/MRI. We classified the lesions regarding PI-RADS version 2.0 and 2.1, peripheral zone (PZ), and transitional zone (TZ), respectively. Based on regions of interest (ROI), standardized uptake values maximum (SUVmax), and corresponding lesion-to-background ratios (LBR) of SUVmax of each category, PI-RADS score 1 to 5, were measured. A comparison between PI-RADS version 2.0 and PI-RADS version 2.1 was performed. Results: A total of 215 focal prostate lesions were analyzed, including two subgroups, 125 TZ and 90 PZ. Data are reported as median and interquartile range (IQR). Regarding PI-RADS version 2.1, TZ SUVmax of each category were 1.5 (0.5, 1.9), 1.9 (0.8, 2.3), 3.3 (2.1, 4.6), 4.2 (3.1, 5.7), 7.3 (5.2, 9.7). PZ SUVmax of each category were 1.0 (0.8, 1.6), 2.5 (1.5, 3.2), 3.3 (1.9, 4.5), 4.3 (3.0, 5.4), 7.4 (5.0, 9.3). Regarding the inter-reader agreement of the overall PI-RADS assessment category, the kappa value was 0.723 for version 2.0 and 0.853 for version 2.1. Conclusion: Revisions of PI-RADS version 2.1 results in variations in lesions classification. Lesions with the PI-RADS category of 3, 4, and 5 present relatively higher intraprostatic PSMA uptake, while lesions with the PI-RADS category of 1 and 2 present relatively lower and similar uptake. Version 2.1 has higher inter-reader reproducibility than version 2.0.

## 1. Introduction

Prostate cancer (PCa) is a common malignant disease in the elderly male population [1], and a percentage of patients with early prostate cancer have metastatic disease. PCa is currently the second leading cause of cancer death in men in the western world, and men have a lifetime probability for PCa of 14%. It is important to be able to determine tumor behavior as well as the diagnosis.

Multiparametric magnetic resonance imaging (mpMRI) is a clinical imaging tool for detecting primary PCa and guiding the subsequent biopsy. MpMRI includes T2-weighted imaging (T2WI), diffusion-weighted imaging (DWI), and dynamic contrast-enhanced MRI (DCE-MRI). Prostate cancer is a type of cancer that can be extremely heterogeneous. Therefore, it is particularly important to accurately evaluate and describe the imaging features of prostate cancer lesions. The American College of Radiology, European Radiology of Uroradiology, and AdMeTech Foundation jointly released the Prostate Imaging Reporting and Data System (PI-RADS) version 2.0 in 2015. PI-RADS 2.0 was a standardized assessment of the probability of clinically significant PCa using prostate mpMRI [2,3]. PI-RADS V2.0 is widely recognized internationally among radiologists and urologists, and is widely used in daily practice and research. Many studies have confirmed the value of PI-RADS V2.0, but as expected, they also have some inconsistencies and limitations, including sub-optimal inter-reader reproducibility, relatively high false-negative rate, and decision-making rules, including the amphibolous evaluation criteria of TZ on T2WI. The detection rate of transitional zone (TZ) tumors is lower than that of peripheral zone (PZ) tumors [2,4,5,6,7,8]. To solve the above problems, the PI-RADS Steering Committee, applying a consensus-based process, is suggested that several modifications have been made to PI-RADS V2.0, remaining the framework for assigning scores to each sequence, and using these scores to achieve at an overall assessment category. Given the limited scope of these updates, the updated version described below is called PI-RADS V2.1. There are a few revisions in this version compared to version 2.0. Experts expect that the clinical use of PI-RADS version 2.1 will improve the variability between readers and further simplify the evaluation of PI-RADS for MRI.

Prostate-specific membrane antigen (PSMA) is a transmembrane glycoprotein related to tumor progression and disease recurrence reported as being overexpressed in prostate cancer cells. Furthermore, it is associated with PCa with higher serum prostate-specific antigen (PSA) levels and a higher Gleason score (GS) [9,10]. Positron emission tomography (PET) images are co-registered with computed tomography (CT) scans that are easily acquired and widely available to provide anatomical information for localization of PSMA-avid lesions. Previous studies suggest that [^68^Ga]Ga-PSMA-11 PET/CT has a high detection rate for prostate tumors, with a sensitivity of 67% to 97%. Koerber et al. [11] and Woythal et al. [12] reported that the standardized uptake values maximum (SUVmax) of PCa is higher than that of noncancerous prostate and healthy prostate tissue. Combining [^68^Ga]Ga-PSMA PET and mpMRI can improve localization accuracy and diagnostic efficiency, as Zamboglou et al. proved [13]. Eiber et al. [14] demonstrated that advances diagnostic accuracy for PCa localization both compared with mpMRI and with PET imaging alone. In the above studies, the advantages of PET/MRI in the diagnosis of PCa have been elaborated.

Our study aimed to (1) evaluate the correlation between PSMA uptake and MRI PI-RADS of the same cohort of prostate focal lesions regarding both PI-RADS version 2.0 and version 2.1, respectively, on MRI, and (2) compare the difference between these two versions.

## 2. Results

### 2.1. Characteristics of Patients

In this study, we analyzed forty-six patients in a total of 215 lesions. Few patients underwent radical prostatectomy (RP) after the scan. The staging is clinical staging based on the physical exam results, prostate biopsy, and imaging tests. Patient characteristics are compiled in Table 1.

### 2.2. Inter-Reader Agreement

Based on the first readout, regarding the inter-reader agreement of the PI-RADS assessment category between the two readers, the kappa value was 0.723, substantial for version 2.0; and 0.853, almost perfect for version 2.1.

### 2.3. Lesion Analysis

Based on the second readout, two readers performed consensus reading to decide the ultimate PI-RADS score of each lesion according to PI-RADS version 2.0 and 2.1, respectively, and PET interpretation. A total of 215 focal prostate lesions were detected by mpMRI and PET, including 125 TZ and 90 PZ. TZ SUVmax and corresponding LBR of SUVmax of each category for version 2.0 and 2.1 are presented in Table 2. PZ SUVmax and corresponding LBR of SUVmax of each category for version 2.0 and 2.1 are shown in Table 3.

## 3. Discussion

PI-RADS version 2.1 has a few revisions about clarifications in interpretation criteria. Turkbey et al. expounded on this revision in detail in the review publication [15]. Revisions include three parts, image data acquisition, clarifications in interpretation criteria, and biparametric MRI. In PI-RADS V2.1 interpretation criteria, some revisions change lesion categories, while other revisions offer a more precise definition of categories to improve diagnostic consistency. In this article, we discussed the revised items in PI-RADS version 2.1 point by point. The correlation between PI-RADS and SUVmax was studied. Moreover, we analyzed these revised items’ impact on [^68^Ga]Ga-PSMA-11 PET/MRI diagnosis. We hope that urologists, radiologists, and nuclear medicine physicians can comprehend the diagnostic criteria changes so that clinical diagnosis can be further developed.

**Figure 1:**
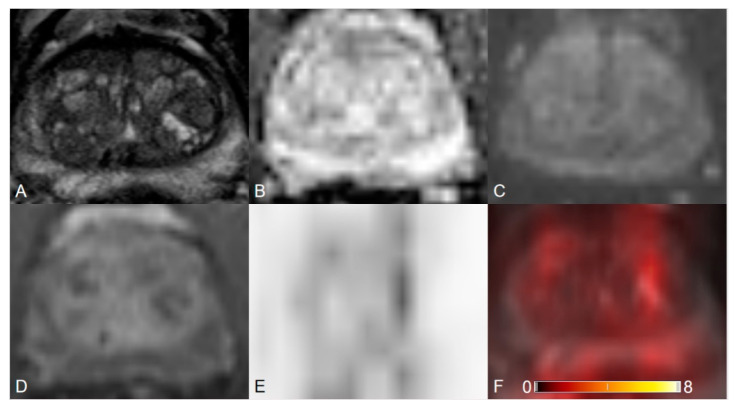
Transition zone with typical benign prostatic hyperplasia (BPH) changes. (**A**) Axial T2WI shows completely encapsulated “typical” nodules. (**B**) ADC map image presents no focal lesion with hypointense signal below the background. (**C**) DWI (b = 1000 s/mm^2^) shows no lesion with a markedly hyperintense signal above the background. (**D**) Early dynamic contrast-enhanced image presents no positive enhancement within the typical BPH nodules. T2WI = 1, DWI = 1, DCE = negative, PI-RADS assessment category = 1. (**E**) PET image shows inhomogeneous [^68^Ga]Ga-PSMA-11 uptake. (**F**) [^68^Ga]Ga-PSMA-11 PET/MRI fusion.

First, for the TZ, T2WI is the primary determining sequence. There is a revision in the criteria for T2WI scores of 1 and 2 in TZ. In PI-RADS V2.0, typical benign prostatic hyperplasia (BPH) nodules, including round, circumscribed, and completely or almost completely encapsulated on T2WI, were assigned a T2WI score of 2. These lesions are assigned a PI-RADS assessment category of 2. In PI-RADS V2.1, a normal-appearing TZ or a round, completely encapsulated nodules are called “typical nodules.” Due to the MRI manifestations of age-related BPH, typical BPH nodules are unlikely to be PCa. In PI-RADS V2.1, findings of BPH alone are considered a normal physiological revision and are assigned a T2WI score of 1. Hence, part of T2WI of 2 lesions revise to T2WI of 1 under the new standard. It is well known that BPH can also show high uptake of PSMA on PET [14,16,17,18]. After a percentage of PSMA-positive BPH nodules are classified as T2WI of 1, then assigned to PI-RADS of 1, the SUVmax of PI-RADS 1 group increases, as shown in Table 2. Figure 1 shows an example of “typical nodules.” Therefore, this remedy will degrade some BPH focal lesions. Doctors may not be obligatory to report such lesions.

**Figure 2:**
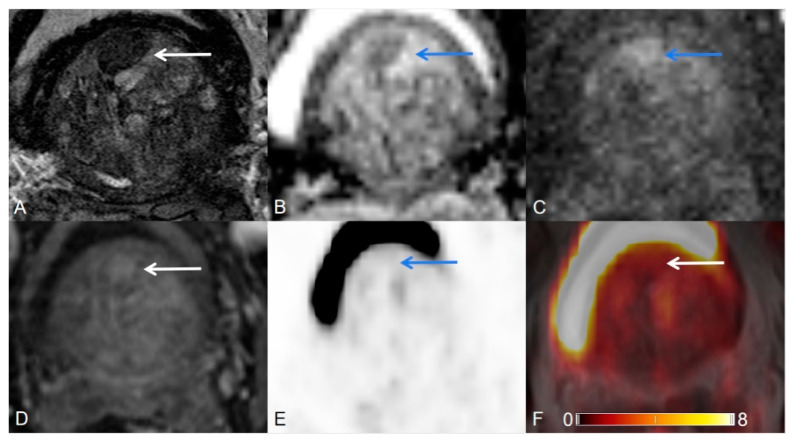
Transition zone with an atypical nodule. (**A**) Axial T2WI shows a homogeneous T2 hypointense, mostly encapsulated nodule. (**B**) ADC map image presents a focal lesion with a markedly hypointense signal below the background corresponding to the lesion seen in (**A**). (**C**) DWI (b = 1000 s/mm^2^) shows a focal lesion with a markedly hyperintense signal above the background corresponding to the lesion seen in (**A**,**B**). (**D**) Early dynamic contrast-enhanced image presents avid enhancement within the nodule. T2WI = 2, DWI = 5, DCE = positive, PI-RADS assessment category = 3. (**E**) PET image shows no [^68^Ga]Ga-PSMA-11 avid uptake. (**F**) [^68^Ga]Ga-PSMA-11 PET/MRI fusion.

Second, there is a revision in the determination of the overall assessment category in TZ. In PI-RADS V2.1, a mostly encapsulated nodule or a homogeneous circumscribed nodule without encapsulation is called “atypical nodules”. The revision in deriving the overall PI-RADS assessment category concerns TZ lesions with a T2WI score of 2, compared to PI-RADS V2.0. In TZ, DWI score of ≥4 now elevates the overall PI-RADS assessment category from 2 to 3 for lesions receiving a T2WI score of 2. DWI score of ≤3 is assigned to PI-RADS of 2 for lesions receiving a T2WI score of 2. Some lesions that initially belonged to PI-RADS 2 are upgraded to PI-RADS 3 under the new standard. Figure 2 shows an example of an atypical nodule with DWI score of ≥4, elevating the overall PI-RADS assessment category from 2 to 3. This revision requires physicians to pay more attention to the information provided by DWI.

**Figure 3:**
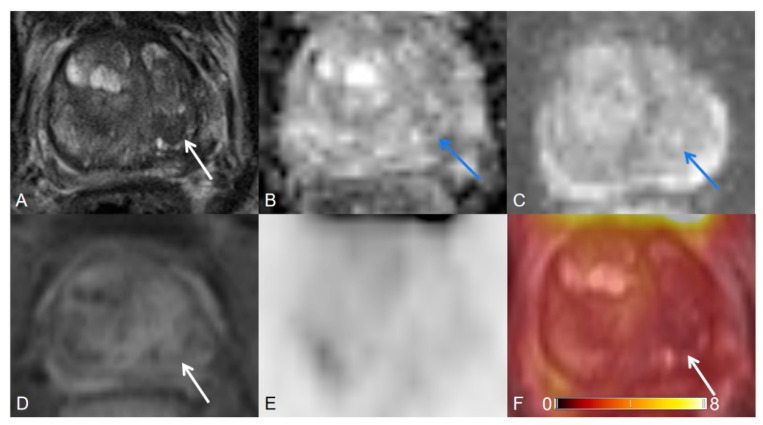
Transition zone with an atypical nodule. (**A**) Axial T2WI shows a T2 hypointense homogeneous circumscribed nodule. (**B**) ADC map image presents a focal lesion with a mildly hypointense signal below the background corresponding to the lesion seen in (**A**). (**C**) DWI (b = 1000 s/mm^2^) shows a focal lesion with a mildly hyperintense signal above the background corresponding to the lesion seen in (**A**,**B**). (**D**) Early dynamic contrast-enhanced image presents no early enhancement within the nodule. T2WI = 2, DWI = 3, DCE = negative, PI-RADS assessment category = 2. (**E**) PET image shows no [^68^Ga]Ga-PSMA-11 avid uptake. (**F**) [^68^Ga]Ga-PSMA-11 PET/MRI fusion.

Furthermore, mildly or moderately restricted diffusion is commonly encountered in mostly encapsulated and unencapsulated TZ lesions. These lesions may represent stromal hyperplasia areas and should not be upgraded based on mildly/moderately restricted diffusion [15]. Lesions with a T2WI score of 1 or 2 should not be upgraded to a PI-RADS assessment category of 2 or 3, respectively, based on a DWI score of 3. Figure 3 shows an example of an atypical nodule with T2WI score of 2, DWI score of 3, and the overall PI-RADS assessment category remains at 2. In PI-RADS V2.1, criteria variations do not affect PI-RADS of 4 and 5 groups. In Table 2, there is no revision in PI-RADS of 4 and 5 groups. Comparing SUVmax and LBR of SUVmax under PI-RADS V2.0 and V2.1, PI-RADS of 1, 2, and 3 groups show a significant difference (*p* < 0.05) between the two versions, while PI-RADS of 4 and 5 show no significant difference (*p* > 0.05) between the two versions.

Third, revision in criteria for DWI scores of 2 and 3. In PI-RADS V2.0, the description of DWI findings score of 2 and 3 is not clear. Due to differentiation in personal understanding, uncertainty and variable interpretation in physicians’ judgment could cause uncertainty when making the diagnosis. Moreover, findings must conform to both apparent diffusion coefficient (ADC) and DWI criteria, but not on just one of the image sets. PI-RADS V2.1 provides a more detailed and clearer definition of DWI findings scores of 2 and 3.

Furthermore, under this new standard, findings could be positive on one of the image sets, ADC, or DWI. This criterion applies to lesions of both TZ and PZ. This revision significantly reduces the diagnosis of uncertainty. For the PZ, DWI is the primary determining sequence and dominant technique. This revision will improve the diagnostic stability of PZ lesions.

Fourth, clarification of the distinction between positive and negative enhancement on DCE. In PI-RADS V2.0, the features that represent a negative DCE score and widespread multifocal enhancement assessment are indefinite. In PI-RADS V2.1, the description for a negative score on DCE has been modified. It can be expected that this revision will reduce the differences among readers in the interpretation of DCE MRI. For the PZ lesions, DCE is the secondary determining sequence. This revision can improve diagnostic reproducibility. Table 3 listed the number of lesions under the two versions PI-RADS. It can be found that there are not so many revisions of PZ. Because for PZ, the revised standard will not make a huge revision in the classification of lesions, but it can improve the inter-reader agreement. From the inter-reader agreement evaluation result, PI-RADS V2.1 shows better consistency between readers than PI-RADS V2.0.

Compared to PI-RADS V2.0, PI-RADS V2.1 is clearer and reduces the diagnosis uncertainty. To sum up the above points, we evaluated the correlation between PSMA uptake and MRI PI-RADS of simultaneous [^68^Ga]Ga-PSMA-11 PET/MRI regarding PI-RADS version 2.0 and 2.1, respectively, and compared the difference between these two versions. We compared the inter-reader reproducibility between PI-RADS version 2.0 and version 2.1. We understand the impact of the reader experience. Generally, the experienced reader achieves a higher detection rate than the inexperienced reader, even though they follow the same PI-RADS criteria. This difference is caused by the different understanding of diagnostic criteria by each doctor. Therefore, a more accurate description of diagnostic criteria is helpful to improve the reproducibility among readers, and this is the aim of the new guideline.

Part of TZ lesions with T2WI score of 2 was upgraded to 3 because the corresponding DWI is ≥4. Part of BPH decreased from PI-RADS assessment category of 2 to 1. Some PI-RADS 2 TZ lesions with avid PSMA uptake were reassigned into other groups. In PI-RADS V2.1, the number of TZ lesions in an overall score of 2 reduced. SUVmax and corresponding LBR also decreased. Although DWI and DCE criteria have revisions, the diagnosis of TZ lesions in PI-RADS 4 and 5 groups is usually very clear and has a high level of reader certainty. PI-RADS V2.1 does not have a significant effect on TZ PI-RADS 4 and 5 groups. There is no revision of SUVmax and corresponding LBR of these two groups. Considering PZ lesions, DWI and DCE are determining sequence. In PI-RADS V2.1, the revisions of DWI and DCE enhance inter-reader agreement.

The variability has been discussed in previous research. Westphalen et al. [19] performed a multi-center study across 26 centers to evaluated variability of the positive predictive value (PPV) of PI-RADS V2.0 for prostate MRI. Across all centers, the estimated PPV was 35% for a PI-RADS score greater than or equal to 3 and 49% for a PI-RADS score greater than or equal to 4. They concluded that the PPV of the PI-RADS V2.0 was low and varied widely across centers.

Some published articles also compared the detection performance of two versions and proved that PI-RADS V2.1 could be preferable for evaluating lesions and achieved a higher inter-reader agreement [20,21,22]. Barrett et al. [23] specifically stated in the review article that the revision of PI-RADS V2.1 is an important step in diagnosing prostate cancer. These studies have reached a common conclusion that PI-RADS V2.1 had better inter-reader reproducibility than did PI-RADS V2.0.

Imaging analysis has been an important tool for the diagnosis and staging of prostate cancer. The present study’s clinical implications help radiologists, nuclear medicine physicians, and urologists understand the essential points in diagnosing and reporting prostate cancer, as well as the revisions in the new standards. Making clinical decisions about patient care is a complex process, which involves processing information and evaluating evidence, while applying critical thinking and problem-solving skills. Optimizing the scoring criteria helps doctors develop personalized treatment plans for patients. PI-RADS has already undergone several revisions. With radiologists and urologists’ joint efforts, the lesion scoring details have become clearer and more precise. Ambiguous descriptions have been revised to make it easier to reach an agreement between radiologists. Multi-modality imaging, [^68^Ga]Ga-PSMA-11 PET/CT, and PET/MRI examination trend toward precision medicine in recent years. It shows encouraging results and is expected to effectively improve the treatment of prostate cancer patients [24,25,26,27]. PET/MRI works based on developing a fusion of PET sequences with MRI sequences for diagnostic purposes in oncological applications. MRI evaluates soft tissue and lymph nodes involvement and bone lesions, while PET provides biological information about cancer. MRI shows superior resolution to CT and PET/CT in the T staging of primary prostate malignancies. An increasing number of researchers have reported that benign lesions and normal tissue show varying degrees of avid PSMA uptake. Therefore, revisions in the PI-RADS will also lead to changes in the correlation between SUVmax and PI-RADS.

In a word, we proved that PI-RADS version 2.1 has comparable performance in detecting prostate focal lesions compared with version 2.0. The results of this study showed that PI-RADS V2.1 can improve the repeatability between readers and may help to improve diagnostic performance and accuracy. Therefore, it is of far-reaching significance for urologists and oncologists to make clinical decisions.

## 4. Materials and Methods

### 4.1. Patients

This retrospective study was approved by the institutional ethics review board (EA1/060/16), and the institutional review board waived the requirement for informed consent for this retrospective analysis.

**Figure 4:**
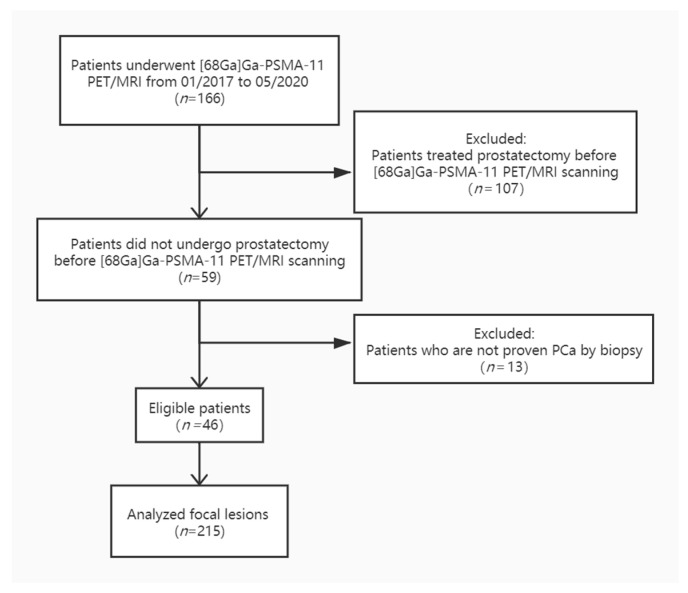
[^68^Ga]Ga-PSMA-11 PET: gallium 68–labeled prostate-specific membrane antigen PET, mpMRI: multiparametric MRI.

Inclusion criteria: (1) patients with biopsy-proven prostate cancer who underwent [^68^Ga]Ga-PSMA-11 PET/MRI between January 2017 and May 2020 in our institute; (2) all necessary additional information could be obtained from our database. Exclusion criteria: (1) patients, who underwent radical prostatectomy before scanning and no prostate left in the pelvic, were excluded; (2) additional information was not adequate. Patient inclusion and exclusion are summarized in the flowchart in Figure 4.

### 4.2. [^68^Ga]Ga-PSMA-11 PET/MRI Imaging Protocol

[^68^Ga]Ga-PSMA-11 was synthesized using a clinical-grade ^68^Ge/^68^Ga radionuclide generator (Eckert & Ziegler Radiopharma GmbH, Berlin, Germany) and PSMA-HBED-CC (ABX GmbH, Radeberg, Germany) as described previously [28,29,30]. Patients were imaged after 85 ± 6 min after intravenous injection of a mean activity of 158.0 ± 18.4 MBq (4.3 ± 0.5 mCi) [^68^Ga]Ga-PSMA-11, corresponding to activity: 1.8–2.2 MBq (0.049–0.060 mCi) per kilogram bodyweight. Furosemide is injected to minimize halo artifact caused by scatter overcorrection associated with high renal and urinary tracer activity 30 min before the start of PET acquisition. Patients were asked to void urine immediately before the start of the examination. No adverse effects were observed after [^68^Ga]Ga-PSMA-11 injection.

Imaging was performed on a 3.0T PET/MRI system (SIEMENS MAGNETOM Biograph mMR, Erlangen, Germany). The acquisition was split into two parts. First, body PET/MRI cover from the vertex to mid-thigh was performed with 3 min of PET acquisition in each bed position, with coverage of 24 cm. Pre-contrast MRI sequences were acquired simultaneously using a combination of a dedicated mMR head-and-neck coil and phased-array mMR body surface coils. Siemens StarVIBE eliminates motion artifacts.

The second part was a dedicated MRI scan of the pelvis, followed by the reconstruction of PET data. MRI sequence parameters are summarized in Table 4. Reconstruction was conducted with an ordered subset expectation maximization algorithm (OSEM), with 3 iterations/21 subsets, based on an x-matrix acquisition with a 4 mm Gaussian filter and relative scatter scaling. Pre-contrast imaging data serve for attenuation correction. PET and MRI were performed using the same protocol for every patient.

### 4.3. Image Analysis

Image analysis was achieved using dedicated post-processing software Syngo.via (Siemens Healthcare, Erlangen, Germany). T2WI was used for anatomic correlation for [^68^Ga]Ga-PSMA-11 PET. Image analysis is comprised of twice readout. The first readout, incorporating both the MRI and PET images as well as all clinical information, were interpreted independently by two double-trained radiologists with 5 and 10 years of experience. Both readers interpreted MRI images using two versions PI-RADS, version 2.0 and 2.1. Then we compared the result of two readers of each version to evaluate inter-reader reproducibility. In the second readout, two readers performed consensus reading to determine the final PI-RADS score of each lesion according to PI-RADS version 2.0 and 2.1, respectively.

ROI were defined as regions with an abnormal signal focal lesion on MRI images or an area with PSMA avid uptake focal lesion on [^68^Ga]Ga-PSMA-11 PET. Readers interpreted mpMRI images according to PI-RADS version 2.0 and version 2.1, respectively. [^68^Ga]Ga-PSMA-11 PET images were interpreted by SUVmax and corresponding LBR, measured based on ROI. LBR is defined as a ratio of lesion SUVmax to background SUVmax. Using ratio value, LBR is to reduce the bias from a specific combination of radiotracer manufacturer, systems vendor, reconstruction techniques, uptake time, post-processing software, the time between radiotracer injection to scanning. Any avid focal lesion in the prostate with uptake above prostate background not attributable to physiologic radiotracer biodistribution was considered positive on [^68^Ga]Ga-PSMA-11 PET. Lesions with the same or lower uptake than the background were deemed negative on [^68^Ga]Ga-PSMA-11 PET. Prostate background SUVmax was measured in the nearest visually defined normal tissue adjacent to a lesion as 1.0 cm^2^, a perfect circle.

### 4.4. Statistical Analysis

We classified the same cohort prostate focal lesions according to PI-RADS version 2.0 and 2.1. SUVmax and corresponding LBR were measured and compared. We divided all lesions into two subgroups, TZ and PZ. All statistical analyses were performed using SPSS 25 for Windows (IBM Corp, Armonk, NY, USA). We evaluated the inter-reader agreement of the PI-RADS assessment category for PI-RADS version 2.0 and 2.1 using the kappa(k) value. Kappa values indicate: poor 0.0, slight 0.0–0.20, fair 0.21–0.40, moderate 0.41–0.60, substantial 0.61–0.80 and, almost-perfect 0.81–1.00 [31]. The Mann–Whitney U-test was used for data comparison between PI-RADS version 2.0 and 2.1.

The significance level was set to two-tailed *p* < 0.05. Patient demographics and clinical characteristics are summarized using descriptive statistics. Normal distributed data are reported as mean ± standard deviation (SD), and non-normal distributed data are reported as median (interquartile range) (IQR Q1, Q3).

## 5. Limitations

There are some limitations to this study. First, this was a retrospective, single-center study. Therefore, the present results may need further validation in prospective multi-center studies with a larger number of patients. Second, this is a descriptive imaging analysis. Due to the characteristics of the patients’ population in our institute, all patients were confirmed PCa by standard biopsy before [^68^Ga]Ga-PSMA-11 PET PET/MRI scanning. MRI-ultrasound fusion-guided prostate biopsy might not be performed, or the information may not be acquirable; therefore, we were not able to study the relationship between PI-RADS scores and targeted biopsy results.

## 6. Conclusions

In conclusion, revisions of PI-RADS version 2.1 results in variations in lesions classification. Lesions with the PI-RADS category of 3, 4, and 5 present relatively higher intraprostatic PSMA uptake, while lesions with the PI-RADS category of 1 and 2 present relatively lower and similar uptake. PI-RADS version 2.1 has higher inter-reader reproducibility than PI-RADS version 2.0. Our result indicated that by using the updated version, radiologists and nuclear medicine doctors are more unlikely to perform an equivocal diagnosis, which could provide more useful information to urologists and oncologists for the management of lesions and clinical decisions making.

## Figures and Tables

**Table 1:** Summary of patient characteristics.

Characteristics	*N* = 46
Age at scan (years)	75 ± 7
PSA (ng/mL) at scan time	12.48 (4.33, 26.48)
Primary tumor stage (*n*)	
≤ cT2c	16 (35%)
≥ cT3a	30 (65%)
Primary lymph node stage (*n*)	
cN0	25 (54%)
cN1	21 (46%)
Biopsy Gleason score (*n*)	
3 + 3	10 (22%)
3 + 4	9 (19%)
4 + 3	7 (15%)
4 + 4	10 (22%)
4 + 5	3 (7%)
5 + 4	5 (11%)
5 + 5	2 (4%)
Treatment	
ADT prior to scan (*n*)	2 (4%)
ADT ongoing at the time of scan (*n*)	9 (19%)
Radiotherapy prior to scan (*n*)	8 (17%)

**Table 2:** TZ lesions SUVmax and corresponding LBR of SUVmax for Version 2.0 and 2.1.

PI-RADS	Version 2.0 (*n*)	SUVmax	LBR of SUVmax	Version 2.1 (*n*)	SUVmax	LBR of SUVmax	SUVmax *p* Value	LBR *p* Value
1	12	1.1 (0.4, 1.7)	1.2 (0.5, 1.9)	21	1.5 (0.5, 1.9)	1.4 (0.7, 2.1)	*p* = 0.02	*p* = 0.02
2	34	2.4 (1.3, 3.2)	2.1 (1.5, 2.9)	21	1.9 (0.8, 2.3)	1.6 (0.9, 2.4)	*p* = 0.02	*p* = 0.02
3	25	3.1 (2.1, 4.4)	2.5 (1.5, 3.5)	29	3.3 (2.1, 4.6)	2.6 (1.5, 3.6)	*p* = 0.73	*p* = 0.84
4	26	4.2 (3.1, 5.7)	3.4 (2.5, 4.8)	26	4.2 (3.1, 5.7)	3.4 (2.5, 4.8)	*p* = 1	*p* = 1
5	28	7.3 (5.2, 9.7)	6.8 (3.3, 12.8)	28	7.3 (5.2, 9.7)	6.8 (3.3, 12.8)	*p* = 1	*p* = 1

Data is presented as median, interquartile range (Q1, Q3). LBR: lesion-to-background ratio.

**Table 3:** PZ lesions SUVmax and corresponding LBR of SUVmax for Version 2.0 and 2.1.

PI-RADS	Version 2.0 (*n*)	SUVmax	LBR of SUVmax	Version 2.1 (*n*)	SUVmax	LBR of SUVmax	SUVmax *p* Value	LBR *p* Value
1	14	1.0 (0.8, 1.6)	1.1 (0.4, 1.8)	14	1.0 (0.8, 1.6)	1.1 (0.4, 1.8)	*p* = 1	*p* = 1
2	15	2.5 (1.3, 3.3)	2.1 (1.5, 2.9)	18	2.5 (1.5, 3.2)	2.2 (1.6, 2.9)	*p* = 0.81	*p* = 0.86
3	18	3.1 (2.0, 4.5)	2.5 (1.5, 3.5)	13	3.3 (1.9, 4.5)	2.6 (1.5, 3.6)	*p* = 0.85	*p* = 0.87
4	18	4.3 (2.9, 5.4)	3.8 (2.7, 4.4)	20	4.3 (3.0, 5.4)	3.8 (2.8, 4.8)	*p* = 0.92	*p* = 0.95
5	25	7.4 (5.0, 9.3)	6.9 (3.1, 11.9)	25	7.4 (5.0, 9.3)	6.9(3.1, 11.9)	*p* = 1	*p* = 1

Data is presented as median, Interquartile range (Q1, Q3). LBR: Lesion-to-background ratio.

**Table 4:** Imaging parameters used for MRI.

Sequence	TR/TE (ms)	FOV (mm)	Flip Angle (degrees)	Section Thickness (mm)	Voxel Size (mm)
T2WI HASTE Axial	1400.0/95.0	400	160	5.0	1.3 × 1.3 × 5.0
T1WI FS VIBE	1600.0/96.0	350	160	4.0	1.1 × 1.1 × 4.0
T2WI Axial	5500.0/103.0	180	150	3.0	0.5 × 0.5 × 3.0
T2WI Sagittal	1600.0/96.0	350	160	4.0	1.1 × 1.1 × 4.0
T2WI Coronal	4500.0/102.0	200	173	3.0	0.4 × 0.4 × 3.0
DWI	11,600.0/70.0	280		3.0	2.5 × 2.5 × 3.0
T1WI FS TWIST dynamic	7.41/3.30	260	12	3.5	1.4 × 1.4 × 3.5
T1WI STARVIBE	3.71/1.77	360	9	1.2	1.1 × 1.1 × 1.2

## Abbreviations

ADC	apparent diffusion coefficient
ADT	Androgen deprivation therapy
BPH	Benign prostatic hyperplasia
CI	Confidence interval
CT	Computed tomography
DCE-MRI	Dynamic contrast-enhanced MRI
DWI	Diffusion-weighted imaging
IQR	Interquartile range
FOV	Field-of-view
Ga	Gallium
GS	Gleason score
LBR	Lesion-to-background ratio
MRI	Magnetic resonance imaging
mpMRI	Multiparametric magnetic resonance imaging
OSEM	Ordered subset expectation maximization algorithm
PCa	Prostate cancer
PET/CT	Positron emission tomography/computed tomography
PET/MR	Positron emission tomography /magnetic resonance
PI-RADS	Prostate Imaging Reporting and Data System
PSA	Prostate-specific antigen
PSMA	Prostate-specific membrane antigen
PROMISE	Prostate Cancer Molecular Imaging Standardized Evaluation
ROI	Region of interest
RP	Radical prostatectomy
SUV	Standardized uptake value
TE	Echo time
TR	Repetition time
T2WI	T2-weighted imaging
